# Tuberculosis Epidemiology and Spatial Ecology at the Cattle-Wild Boar Interface in Northern Spain

**DOI:** 10.1155/2023/2147191

**Published:** 2023-02-23

**Authors:** Gloria Herrero-García, Pelayo Acevedo, Pablo Quirós, Miguel Prieto, Beatriz Romero, Javier Amado, Manuel Antonio Queipo, Christian Gortázar, Ana Balseiro

**Affiliations:** ^1^Departamento de Sanidad Animal, Facultad de Veterinaria, Universidad de León, León 24071, Spain; ^2^SaBio-IREC (CSIC-UCLM), Ciudad Real 13071, Spain; ^3^Gobierno del Principado de Asturias, Oviedo 33005, Spain; ^4^Servicio Regional de Investigación y Desarrollo Agroalimentaria del Principado de Asturias-SERIDA, Gijón 33394, Spain; ^5^Centro de Vigilancia Sanitaria Veterinaria and Departamento de Sanidad Animal, Facultad de Veterinaria, Universidad Complutense de Madrid, Madrid 28040, Spain; ^6^Departamento de Sanidad Animal, Instituto de Ganadería de Montaña (CSIC-Universidad de León), Finca Marzanas, Grulleros 24346, León, Spain

## Abstract

Tuberculosis (TB) is a contagious chronic disease due to infection with *Mycobacterium tuberculosis* complex (MTC) bacteria. Monitoring of wildlife, especially potential reservoirs, is important for detecting changes in disease occurrence and assessing the impact of interventions. Here, we examined whether wild boar (*Sus scrofa*) may contribute to the re-emergence of TB in Asturias (10,604 km^2^), northern Spain. Although this province was declared free of TB in cattle in November 2021, MTC bacteria remain prevalent in several “hotspots,” with the European badger (*Meles meles*) suggested as a TB potential wild reservoir. Drawing on data from the Spanish National Bovine Tuberculosis Eradication Program and the Government of the Principality of Asturias covering the period 2014–2020, we analyzed the prevalence of TB in cattle and wild boar in this region. In hotspots (592 km^2^), we also investigated the ranging behavior and habitat use of five cows that belonged to farms with a history of TB and six trapped sympatric wild boar. During the observation period, TB prevalence was 0.14% among cattle overall and 0.13–0.41% in hotspots, which was much lower than the prevalence in wild boar, which was 3.15% overall and 5.23–5.96% in hotspots. Infected cattle and infected wild boar in hotspots shared the same strains of *M. bovis*, and GPS tracking showed spatiotemporal overlap between the species, mainly around pastures during sunrise (06:00–07:00 h) and sunset (19:00–20:00 h). Our results suggest that in addition to cattle and badgers, wild boar possibly help maintain TB in northern Spain, increasing the host richness that influences TB transmission risk in the area, which should be taken into account in monitoring and eradication efforts.

## 1. Introduction

Tuberculosis (TB) is a contagious chronic disease due to infection with *Mycobacterium tuberculosis* complex (MTC) bacteria, principally *M. bovis* and *M. caprae* [1]. The disease is a major social, economic, and public health challenge, affecting domestic and wild animals [2]. Reservoirs of the disease among wildlife can reduce the efficacy of efforts to eradicate it from cattle [3]. TB in Europe affects multiple species, such that most infected animals are not bovine [4], implying a wide range of potential reservoirs. For example, the native Eurasian wild boar (*Sus scrofa*), whose populations are growing in Europe [5, 6], can contribute substantially to TB epidemiology in southwestern Spain and France [7–9]. On the other hand, the European badger (*Meles meles*) is the reservoir of TB in the British Islands [10]. The influence of these and other wild TB reservoirs depends on numerous factors, including the prevalence of TB among cattle, characteristics and density of reservoir population, land use, as well as cattle and wildlife management practices [11–13].

The abundance of potential TB reservoirs in the wild highlights the need for integrated wildlife monitoring in order to identify changes in disease occurrence and assess the impact of interventions [14–16]. This is particularly true in “hotspots” where MTC bacteria have become endemic and remain prevalent in cattle [17]. Such monitoring should examine the epidemiology and temporal dynamics of TB [18, 19] as well as the spatial ecology of wildlife [11, 20]. A particularly useful tool in these efforts is the global positioning system (GPS) technology, which can monitor individuals and characterize the network of interactions within and between potential hosts to clarify how pathogens persist and spread between livestock and wildlife [21–24].

Asturias (10,604 km^2^), in northern Spain, was declared free of TB in cattle in November 2021, Implementing Regulation (UE) 2021/1911 [25], but several hotspots persist, and the region appears to contain potential disease reservoirs, i.e., badger [26]. Disease transmission between badgers and cattle has been documented and supported by, for example, GPS studies showing badgers' presence in cattle paddocks [20, 26]. In this way, Asturias is an excellent example of a region where a comprehensive understanding of TB persistence and transmission in multiple wild hosts is essential to control the disease.

To clarify to what extent the interaction between wild boar and cattle helps maintain TB in this area, we analyzed disease prevalence in these species in hotspots in Asturias during the period 2014–2020. We also used GPS collars to track the ranging behavior and habitat use of wild boar in relation to cattle. Our results may help clarify how wild hosts can maintain TB in endemic hotspots within an area declared free of the disease in cattle.

## 2. Materials and Methods

### 2.1. Ethical Statement

All methods were carried out in accordance with relevant guidelines and regulations [27]. All experimental protocols were approved by ethical committees from the Government of Principality of Asturias, license reference number: PROAE 47/2018.

### 2.2. Study Area

**Figure 1:**
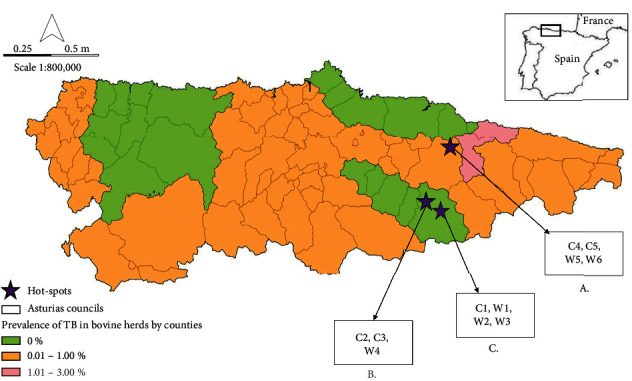
Study area. Location of the study area (Asturias) in northwestern Spain, delimitation of counties, situation of the hotspots of Piloña (*n* = 1; A) and Caso (n = 2; B and C), monitored animals in each hotspot [*n* = 5 cows (C1, C2, C3, C4, C5); *n* = 6 wild boar (W1, W2, W3, W4, W5, W6)], and 2020 TB prevalence situation in bovine herds in Asturias (information obtained from [25]).

The study was carried out in three TB hotspots in Piloña (A) and Caso (B, C) in Asturias (Figure 1), which are located in northwestern Spain (43°21′ N; 5°57′ W). This region is characterized by an Atlantic climate, abundant precipitations (>1000 mm/year), soft temperatures, and small thermal amplitude [28]. The land varies from 0 m to 2650 m above sea level (“Torrecerredo” peak) and is covered by 1.8% of cultures, 29.4% of pastures and 57.5% of forested land, approximately [29]. Here, large-scale fencing for cattle does not exist, and wild boar, whose abundance has increased in the last years, moves freely over the territory [30].

### 2.3. Tuberculosis Prevalence in Cattle and Wild Boar

Cattle TB prevalence data from 2014 to 2020 for Asturias and Caso and Piloña (see Figure 1) were obtained from the 2022 National Bovine Tuberculosis Eradication Program [25], whilst wild boar TB prevalence data were obtained from the Government of the Principality of Asturias. Data were obtained by the official single intradermal tuberculin test and culture in cattle and by culture in wild boar (*n* = 1341).

In this study, cattle (*n* = 12) and wild boar (*n* = 29) MTC isolates from Caso and Piloña during this period were submitted for MTC species identification by PCR and subsequent mycobacterial interspersed repetitive units-variable number of tandem repeats (MIRU-VNTR) typing. A quantitative PCR was performed on culture isolates, in which the MTC forward-primer 5′-TAGTGCATGCACCGAATTAGAACGT-3′ and the MTC reverse primer 5′-CGAGTAGGTCATGGCTCCTCC-3′ were used, in addition to the TaqMan probe YY/BHQ 5′- AATCGCGTCGCCGGGAGC-3′, which amplifies a 184-bp fragment [31]. MTC isolates were characterized using DVR-spoligotyping (VISAVET, Madrid, Spain) and coded according to the *M. bovis* spoligotype database website [32]. To confirm similarity between the isolates from both species, MIRU-VNTR typing was performed using the following nine VNTR markers: ETR-A, ETR-B, ETR-D, ETR-E, MIRU26, QUB11a, QUB11b, QUB26, and QUB3232, as described previously [33].

Differences in prevalence between species were assessed using Mann–Whitney *U* tests. The statistical test was carried out using SPSS Statistics 25 (IBM, New York, USA), and the significance level was set at *p* < 0.05.

### 2.4. Animal Trapping and Monitoring

Different cattle and wild boar were monitored in relation to their ranging behavior in Caso and Piloña (approximately an area of 592 km^2^) (Figure 1).

#### 2.4.1. Cattle

Five adult cows (C1, C2, C3, C4, and C5) were monitored in the three hotspots (see Figure 1). We randomly selected cows that belonged to farms with a history of TB. They were tracked using Digitanimal GPS radio-collars (Digitanimal®, Madrid, Spain), programmed to acquire one location per 30 min, including time, date, geographic coordinates, and temperature (Table 1).

#### 2.4.2. Wild Boar

Six adult free-ranging wild boar were monitored in the three hotspots (W1, W2, W3, W4, W5, and W6) (see Figure 1). Wild boar showing good body condition were randomly selected. Homologated cages (Jauteco®, Spain) were used to trap the animals, after which they were anaesthetized with tiletamine-zolazepam (0.06 mL/kg) and ketamine (0.02 mL/kg), administered by means of intramuscular injection [34].

They were monitored in different years and months by using Microsensory GPS radio-collars (Microsensory System, Córdoba, Spain) and Digitanimal GPS radio-collars, programmed to provide the location of the animal in a determined frequency, time, date, geographic coordinates, and temperature (Table 1).

GPS radio-collars in cattle were used for further studies, and they had a shorter programmed acquisition time and a larger monitoring period; consequently, the number of cattle locations was higher than that of wild boar.

### 2.5. Data Analyses

For the 11 monitored animals (cattle and wild boar), activity patterns, home ranges (HRs), and habitat selection patterns were completed. Spatial overlap, environmental overlap, and temporal overlap between species were analyzed to describe the potential of species interactions.

#### 2.5.1. Activity Pattern

To calculate the animal's activity pattern, a straight line was obtained between consecutive GPS locations separated by intervals of 2 hours (h), as locations of wild boar were programmed to acquire information every 2 h (W4 and W5 were not considered due to the lack of locations, see Table 1). This distance was then divided by the time elapsed between them (km/h) and was used to infer activity patterns.

#### 2.5.2. Temporal Overlap

Overlap analysis for cattle and wild boar activity patterns acquired was undertaken using the “overlap” R package [35]. Overlap coefficients (Δ) went from 0 (no overlap) to 1 (full overlap), and the 95% confidence intervals (CI) were obtained through 1000 bootstrap samples. As all samples were >50 records, the coefficient Δ4 was considered [36].

#### 2.5.3. Home Range

Annual and seasonal HRs were estimated using the fixed-kernel function in the “adehabitat” R package, R version 3.6.1 in [35] program [37]. Kernel 95% was used to indicate the home range (HR), and kernel 50% the core range (CR) [38]. The least-squarescross-validation method failed to converge in animals with large sample sizes; therefore, kernels were estimated using the reference bandwidth method [39, 40]. According to previous studies and to estimate HRs [20, 40], the minimum number of relocations per individual was established at 25.

Differences in HR and CR among seasons and species were assessed using Kruskal–Wallis *H* tests and Mann–Whitney *U* tests, respectively.

#### 2.5.4. Spatial Overlap

Home range and CR were used to estimate spatial overlap between wild boar and cattle within the study area. Spatial overlap was calculated using the overlap function in “rgeos” R package [35]. This gave information on the area intersected (HR and CR) between cattle and wild boar relative to cattle when the area was divided by the HR or CR of the wild boar and vice versa when it was divided by the HR or CR of the cattle [41].

#### 2.5.5. Environmental Overlap

Land uses of Caso and Piloña were obtained from a combination of the Corine Land Cover [42] and the National Center for Geographic Information [43], with scale cartography of 1:250,000 and considering the following land uses: cultures, pastures, woodland, shrubland, water, no vegetation areas, and urban areas, as seen in previous studies [20, 44, 45], in view of biological relevance. In this study, urban areas were villages with low population density (<27 inhab/km^2^), with economical activities mainly based on agricultural and livestock production.

GPS locations were buffered to assess interactions between cattle and wild boar in different land uses. These considered GPS positional error and missing locations to avoid misclassification of habitat use, possibly given by landscape heterogeneity and lack of GPS locations [46]. The proportional cover of each land use was calculated within each buffer. Latent selection difference functions (LSDs) were estimated using logistic regression and the “rms” R package [35], as described in Barasona et al. [11]. In these analyses, locations of cattle were coded as 1 and locations of wild boar as 0, so cattle resource selection or avoidance relative to wild boar could be evaluated. Variables with significant positive coefficients showed preferred land uses by cattle relative to wild boar, and those with significant negative coefficients indicated avoided land uses. However, variables with no significant coefficients indicated areas with no difference of use and, therefore, with a high potential for interaction.

The Huber–White sandwich estimator was used to estimate standard errors, grouping data by the individual, which considered an unbalanced sampling design and nonindependence of observations belonging to the same individual [47]. The best model was considered by means of a forwards-backwards stepwise procedure based on Akaike information criteria (AIC) [48].

## 3. Results

### 3.1. Tuberculosis Prevalence in Cattle and Wild Boar

**Figure 2:**
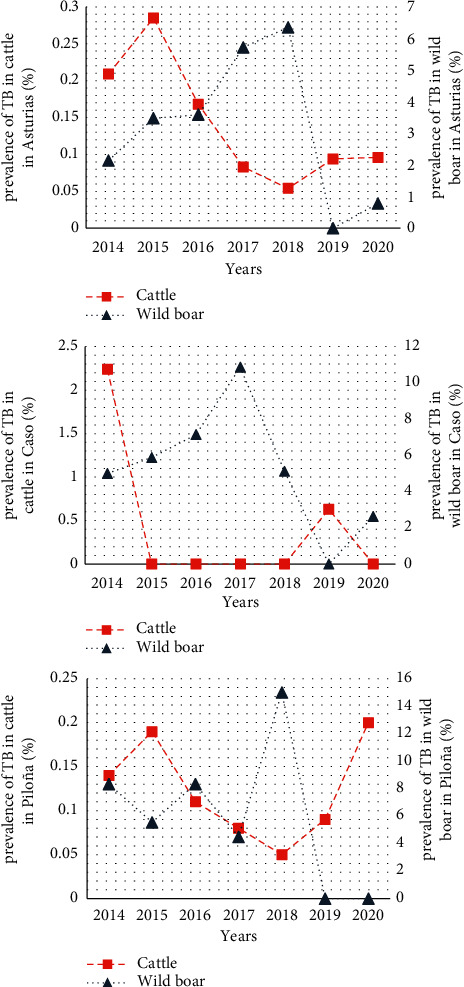
Cattle and wild boar tuberculosis (TB) prevalence trends. Information is given for the region of Asturias and the regions of Caso and Piloña (where the hotspots remain) during 2014–2020.

The prevalence of TB in cattle in Asturias decreased between 2014–2018 [0.21% (2014), 0.28% (2015), 0.17% (2016), 0.08% (2017), and 0.05% (2018)], and showed a slight increase in 2018–2020 [0.05% (2018), 0.09% (2019), and 0.09% (2020)] (Figure 2). Wild boar TB prevalence, however, oscillated in the same period observing the highest prevalence in 2018 [2.14% (95% CI = 0.26%–4.54%; 3/140) in 2014, 3.48% (95% CI = 1.24%–5.72%; 9/258) in 2015, 3.59% (95% CI = 1.59%–5.59%; 12/334) in 2016, 5.72% (95% CI = 2.44%–9.0%; 11/192) in 2017, 6.35% (95% CI = 2.72%–9.98%; 11/173) in 2018, 0.0% in 2019, and 0.78% (95% CI = 0.75%–2.31%; 1/127) in 2020] (Figure 2). Significant differences were observed between species (*p*=0.024).

In Caso, cattle TB prevalence decreased between 2014–2018 and had an upturn in 2019: 2.24% (2014), 0% (2015, 2016, 2017, and 2018), 0.63% (2019), and 0% (2020). In this area, wild boar TB prevalence was higher than that observed for the species in overall Asturias: 5.0% (95% CI = 4.55%–14.55%; 1/20) in 2014, 5.88% (95% CI = 2.03%–13.79%; 2/34) in 2015, 7.14% (95% CI = 0.40%–13.88%; 4/56) in 2016, 10.87% (95% CI = 1.87%–19.85%; 5/46) in 2017, 5.13% (95% CI = 1.80%–12.04%; 2/39) in 2018, 0.0% (0/44) in 2019, and 2.63% (95% CI = 2.46%–7.72%; 1/38) in 2020 (Figure 2). Significant differences were observed between species (*p* = 0.008).

In Piloña, the TB prevalence of cattle was as follows: 0.14% (2014), 0.19% (2015), 0.11% (2016), 0.08% (2017), 0.05% (2018), 0.09% (2019), and 0.20% (2020). In that region, wild boar TB prevalence by years also showed higher percentages than for Asturias: 8.33% (95% CI = 2.73%–19.39%; 2/24) in 2014, 5.41% (95% CI = 1.83%–12.93%; 2/37) in 2015, 8.33% (95% CI = 2.73%–19.39%; 2/24) in 2016, 4.49% (95% CI = 0.19%–8.79%; 4/89) in 2017, 15.0% (95% CI = 3.93%–26.07%; 6/40) in 2018, 0% (0/14) in 2019, and 0% (0/25) in 2020 (Figure 2). No significant differences were observed between species in Piloña (*p*=0.195).

Both in Caso and Piloña, the same *M. bovis* isolates were characterized in cattle and wild boar from the same region. The identified 4 spoligotypes and VNTR profiles are shown in Table 2.

### 3.2. Cattle and Wild Boar TB Epidemiology

#### 3.2.1. Activity Pattern and Temporal Overlap

**Figure 3:**
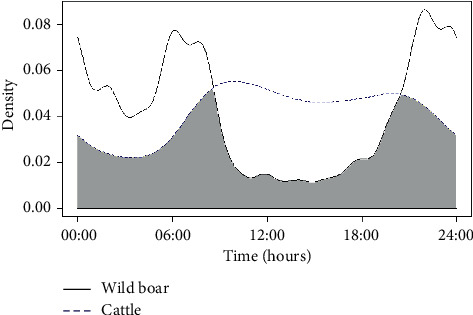
Cattle and wild boar activity patterns and overlap among them. Animals were monitored in the three hotspots, and activities were measured for summer. Overlap (gray shade) between cattle's activity pattern (blue dotted line) and wild boar's activity pattern (black line) among the different hours of the day is indicated.

To compare activity patterns of cattle and wild boar, and due to the absence of information on wild boar GPS monitoring in autumn, winter, and spring, activity patterns were only obtained for summer. Cattle manifested one peak at 06:00 h and a similar movement until 20:00 h, when activity started to decrease. Wild boar, on the other side, exhibited two different peaks in their activity: first at around 05:00 h and second at around 21:00 h, coinciding with sunrise and sunset approximately (Figure 3). However, considering both species, two different periods could be established: (1) from 08:00 h to 20:00 h and (2) from 20:00 h to 08:00 h. The coefficient of overlap (Δ4) resulted in 0.63 (confidence intervals 0.54–0.73) (Figure 3).

#### 3.2.2. Home Range

**Figure 4:**
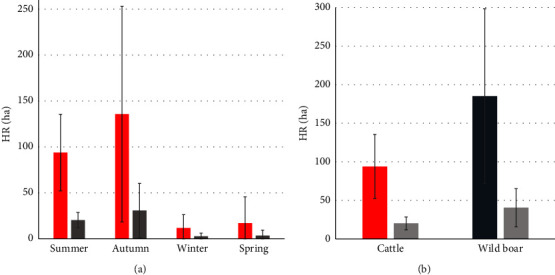
Cattle and wild boar home range (HR) and core range (CR). (a) HR (red) and CR (gray) of cattle in summer, autumn, winter, and spring. (b) HR of cattle (red) and wild boar (dark blue) and CR (gray) for both species in summer. Error bars indicate standard deviations.

Although it seems that wild boar used larger areas than cattle (Figure 4), no significant differences were observed among cattle summer HR and CR sizes (average ± SE, HR = 93.87 ± 42; CR = 20.25 ± 8) and wild boar HR and CR sizes (HR = 185.23 ± 113; CR = 40.50 ± 25) (Mann–Whitney U, *p* = 0.178; *p* = 0.170, respectively). No significant differences were found among the three hotspots.

The largest cattle HR and CR sizes were observed in autumn (HR = 135.73 ± 118; CR = 30.85 ± 29), followed by summer (HR = 93.87 ± 42; CR = 20.25 ± 8), spring (HR = 16.93 ± 29; CR = 3.44 ± 6), and winter (HR = 11.69 ± 15; CR = 2.61 ± 3) (Figure 4). Significant differences were found in cattle HR and CR among seasons (Kruskal–Wallis *χ*^2^ = 9.817, *p* = 0.020; *χ*^2^ = 9.307, *p* = 0.025, respectively).

#### 3.2.3. Spatial Overlap

**Figure 5:**
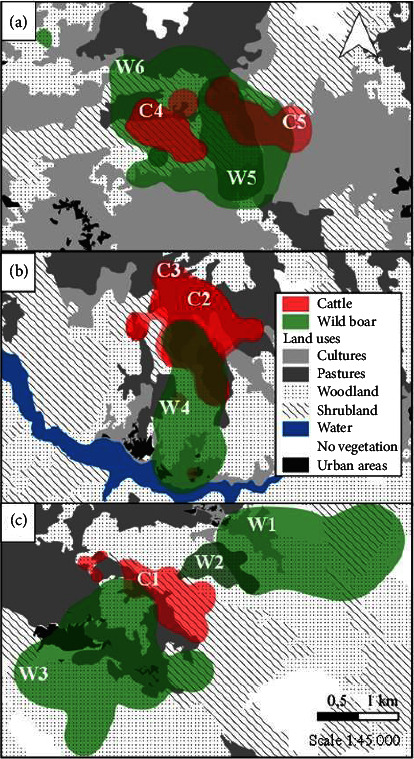
Cattle and wild boar home range (HR). Representation of spatial overlap between cattle HR (in red) and wild boar HR (in green) in the three hotspots: (a) Piloña, (b) and (c) Caso. Information is given for summer. Land uses are included, as well as identification of each of the eleven monitored animals (cattle: C1, C2, C3, C4, and C5, and wild boar: W1, W2, W3, W4, W5, and W6).

Spatial overlaps between cattle and wild boar in HR and CR differed among areas during summer, observing the highest overlap in the hotspot of Piloña (A), followed by hotspot “B” and hotspot “C” in Caso (Figure 5). Overall, >32% of the cattle HR overlapped wild boar HR, whereas around 17% of the wild boar HR overlapped cattle HR. When comparing summer wild boar HR to the annual HR sizes of cattle, overlaps increased in most of them, either HR or CR. In this case, overall, >40% of the cattle HR overlapped with wild boar HR, whereas 28% of the wild boar HR overlapped with cattle HR (Table 3).

#### 3.2.4. Environmental Overlap

Models selected woodland and pastures as areas that better segregated cattle and wild boar (coefficients resulted significant or marginally significant), whereas cultures, shrubland, and urban areas as land use which worst segregated both species (not significant coefficients). Cattle showed avoidance of areas with a higher proportion of woodland, relative to wild boar, and preferred areas with a higher proportion of pastures, also relative to wild boar (Table 4).

The model selected, which better explained the habitat selection of cattle relative to wild boar, chose as influential variables woodland, urban areas, pastures and shrubland. The resulting model was significant (*p* = <0.0001) and with an *R*^2^ = 0.671.

## 4. Discussion

Our 7-year analysis of Asturias indicates that the prevalence of TB among wild boar, particularly in hotspots, has gradually increased, and we have provided evidence that the disease moves between wild boar and cattle. These findings establish wild boar, together with badger [26], as wild reservoirs of the disease in hotspots, which helps clarify how TB remains a threat to cattle in these areas.

The same *M. bovis* strains and VNTR profiles were identified from infected wild boar and infected cattle in our study. Given that TB prevalence among cattle in Asturias increased from 2014 to 2015 and then increased among wild boar from 2016–2018, we speculate that TB might have travelled from cattle to wild boar during the study period. Indeed, an initial increase in disease prevalence among cattle followed by an increase among wild boar was observed in the hotspots of Caso and Piloña. The slight increase in TB prevalence among cattle from 2018 (0.05%) to 2019-2020 (0.09%), in turn, suggests that either other infected cattle or wildlife (i.e., wild boar and badger) might be the source of infection in TB-free farms. Wild boar in Europe have been shown to transmit TB efficiently to cattle [49]. In addition, the population density and distribution of wild boar have increased in northern Spain since the 1990s [30, 50]. The wild boar may continue to grow as a TB threat to cattle in this area, given their ability to thrive in a wide range of habitats [51].

The cattle in our study were active mainly in the middle of the day, whereas wild boar were active mainly in the early morning and late afternoon. Nevertheless, we identified two periods when both species were highly active: 06:00–07:00 h and 19:00–20:00 h. At these times, the two species likely came into contact, given the spatial overlap in their distribution. Thus, restricting cattle movements at sunrise and sunset may help protect them from infected wild boar. Such measures may be particularly important in the hotspot of Piloña, where the two species showed the greatest spatial overlap and the highest TB prevalence among wild boar (2018).

The HR and CR of cattle in our study were significantly larger in autumn and summer than in spring and winter, consistent with the extensive production systems common in Asturias [52]. In such systems, animals are kept in communal pastures and open meadows in spring and summer, with short periods of stabling during winter. In summer, the HR and CR of wild boar were even larger than those of cattle. As cattle and wild boar share habitat use with no or little restrictions in these traditional communal pastures, different herds, as well as wildlife species, can circulate among the same pastures, increasing the risk of disease transmission. Our analysis, therefore, strengthens the case for biosecurity measures in this region [24, 53].

We analyzed the HR of wild boar only during summer, neglecting the hunting season from September to February. During the summer, this HR substantially overlapped with the HR of cattle, implying that the two HRs overlapped to at least some extent during other seasons, including when cattle were in paddocks. Indeed, the HR of wild boar in Asturias remained large throughout the year, though it increased significantly during the hunting season [54]. We speculate that wild boar in Asturias may reach even larger numbers of cattle in the autumn than in the summer. On the other hand, hunting can substantially reduce the number of wild boar [30, 55, 56]. In fact, an efficient hunting season may explain the lower TB prevalence among wild boar in 2018-2019, although influence from differences in field sampling and diagnostic testing cannot be excluded [57]. Future work should consider the effects of hunting season on the TB risk that wild boar poses to cattle. In addition to hunting by humans, predation by gray wolves (*Canis lupus*) can reduce wild boar populations, especially the numbers of TB sickly animals, which are more likely to shed pathogen into the environment, and the numbers of piglets, which are more susceptible to infection. Such predation of wildlife reservoirs has been proposed as a major form of natural infection control [58, 59].

While wildlife reservoirs are a major source of infection for cattle [60, 61], other factors also contribute to infection risk, including the size of the cattle herd, the number of incoming animals in recent years, pasture lease agreements and transhumance to areas with high TB prevalence [62–64]. Future work should explore the full range of factors driving TB prevalence among cattle in Asturias, which may help clarify disease maintenance in other TB-free areas. Such work should carefully consider additional factors that may influence the risk of disease transmission directly or indirectly. For example, although our study reflects that cattle prefer pastures and wild boar prefer woodlands because they provide shelter [45], the cattle dung on pastures favors the presence of earthworms [65], which are known to attract wild boar [66, 67] and also badgers [20], potentially increasing interactions and infection transmission. At the same time, wild boar is expanding into more humanized landscapes, usually when their nutritional needs are not met [68]. Aside from landscape type and land use, environmental contamination may be important for maintaining TB in an area, given that *M. bovis* can persist in favorable environments for long periods [69–71].

Our work underscores that TB in some regions of Europe is a truly multi-host disease that involves cattle as well as domestic and wild nonbovine species [4]. Host richness is an important factor influencing the transmission risk of infectious diseases, including TB [72]. TB in northern Spain is known to be maintained jointly by cattle and badger [26], to which the present work adds wild boar. The dynamics of infection among all these epidemiologically relevant hosts should be considered if TB control programs are to be effective [4, 72].

Our findings in this study, however, should be interpreted with caution as the results in activity pattern, and temporal, spatial, and environmental overlap may be biased due to insufficient animals, lack of locations and short periods of monitoring, thus representing a conservative figure of the epidemiology and spatial ecology in the area.

## 5. Conclusions

Our work shows evidence of spatiotemporal overlap between cattle and wild boar in areas with high TB prevalence, mainly around pastures during sunrise and sunset. We also indicate a possible interspecies TB transmission, likely from cattle to wild boar, although TB prevalence trends suggest that TB-free cattle could also be infected by wild boar. Therefore, in addition to cattle and badger, wild boar can help maintain TB in northern Spain, increasing the host richness that influences TB transmission risk in the area.

## Figures and Tables

**Table 1:** Information provided by the GPS of the monitored animals. Identification given to each animal, months and years in which the GPS provided information, acquisition programmed time (frequency of locations), number of days with data, and number of locations given. Note that the number of cattle locations is higher than the number of wild boar locations due to shorter programmed acquisition time and larger monitoring period.

	ID										
	C1	C2	C3	C4	C5	W1	W2	W3	W4	W5	W6
Month	May–Dec	Jan–Dec	Jan–Oct	Jul–Sep	May–Sep	Jun–Jul	May-Jun	May–Jul	Jul–Sep	Jun–Sep	May–Sep
Year	2020	2020	2020	2020	2020	2020	2020	2020	2019	2018	2019
Progr	30 min	30 min	30 min	30 min	30 min	2 h	30 min	2 h	4 h	4 h	2 h
Days	177	348	234	158	136	19	14	49	50	39	138
Loca	5868	11880	6664	2251	3167	71	246	260	186	167	705

ID: Identification; C: Cow; W: Wild boar; Progr: Programmed time, Loca: Locations, Jan: January, Jun: June, Jul: July, Sep: September, Oct: October, and Dec: December.

**Table 2:** *Mycobacterium bovis* isolates characterized for cattle and wild boar in the tuberculosis hotspots from Caso and Piloña. Spoligotype (SB) number, VNTR profile, number (*n*) of cattle and wild boar, and total of animals characterized are indicated.

	Spoligotype	VNTR profile	Cattle (*n*)	Wild boar (*n*)	Total (*n*)
Caso	SB0134	6-3-3-3-5-9-4-5-5	6	21	27
	SB0121	4-4-3-3-5-10-2-5-8	1	4	5
	SB1658	6-2-3-3-4-9-3-5-5	0	2	2

Piloña	SB0828	5-5-3-4-5-9-3-3-6	5	2	7

**Table 3:** Home range (HR) and core range (CR) spatial overlaps. Percentages of overlap between cattle and wild boar in summer and annual HR and CR. Overlaps are given for the hotspots of Piloña (A) and Caso (B and C).

	Between-species overlap (%)			
	A	B	C	Overall
Cattle relative to wild boar in summer (HR; CR)	(**56.35;** 9.32)	(37.01; 2.05)	(2.89; 0)	(32.1; 3.8)
Wild boar relative to cattle in summer (HR; CR)	(16.54; 8.94)	(33.67; 1.76)	(1.13; 0)	(17.1; 3.6)
Cattle relative to wild boar, annually (HR; CR)	(56.29; 10.01)	(**56.78**; 42.99)	(8.17; 1.72)	(40.41; 18.24)
Wild boar relative to cattle, annually (HR; CR)	(32.29; 8.31)	(47.58; 9.22)	(4.66; 0.79)	(28.17; 6.11)

Bold values indicate highest percentages of spatial overlap between species.

**Table 4:** Results of the model selected. Model coefficients, standard errors (SE), Wald *Z* value, and *p* value.

	Model			
	Coefficient	SE	Wald *Z*	*p*
Intercept	0.7652	0.9548	0.80	0.4229
Woodland	−0.0532	0.0082	−6.46	<0.0001
Urban areas	−0.4797	0.2932	−1.64	0.1019
Pastures	0.0227	0.0119	1.91	0.0560
Shrubland	0.0061	0.0112	0.55	0.5846

## Data Availability

The data that support the findings of this study are available by the corresponding author upon reasonable request.
